# Attentional bias for sad facial expressions in adults with a history of peer victimization

**DOI:** 10.3389/fpsyg.2023.1127381

**Published:** 2023-03-06

**Authors:** Klara Blauth, Benjamin Iffland

**Affiliations:** Department of Psychology, Bielefeld University, Bielefeld, Germany

**Keywords:** peer victimization, child maltreatment, attentional bias, emotional facial expressions, dot-probe task

## Abstract

**Introduction:**

Previous research has indicated altered attentional processing in individuals with experiences of maltreatment or victimization in childhood and adolescence. The present study examined the impact of child and adolescent experiences of relational peer victimization on attentional processes in adulthood when confronted with emotional facial expressions.

**Methods:**

As part of an online study, a community sample of adults completed a facial dot-probe task. In the present task, pictures of facial expressions displaying four different emotions (anger, disgust, happiness, and sadness) were used.

**Results:**

The results of the hierarchical regression analyses showed that retrospective reports of peer victimization made a significant contribution to the prediction of facilitated orienting processes for sad facial expressions. Experiences of emotional child maltreatment, on the other hand, made a significant contribution to the prediction of attentional biases for angry facial expressions.

**Discussion:**

Our results emphasize the relevance of experiences of emotional and relational maltreatment in childhood and in adolescence for the processing of social stimuli in adulthood. The findings regarding emotional child maltreatment are more indicative of attentional biases in the context of threat detection, whereas the altered attentional processes in peer victimization are more indicative of mood-congruent biases. These altered processes may be active in social situations and may therefore influence future social situations, behavior, feelings, and thus mental health.

## 1. Introduction

There are a variety of studies demonstrating the negative impact of maltreatment experiences in a peer context in childhood and adolescence on psychosocial adjustment and particularly mental health (for a review see McDougall and Vaillancourt, 2015). These experiences, also called peer victimization experiences, include different kinds of maltreatment experiences that occur in interactions with peers, e.g., overt forms like physical or verbal violence, or relational maltreatment experiences associated with rejection or exclusion from a social group (De Los Reyes and Prinstein, 2004; Siegel et al., 2009; Sansen et al., 2015). Thus, peer victimization can be distinguished from child maltreatment, where the violence is perpetrated by adults or caregivers, including forms of emotional, physical, or sexual maltreatment (World Health Organization, 1999). Similar to the negative effects of child maltreatment (for a review see Carr et al., 2020), peer victimization is associated with problems in several areas, such as academic achievement or social adjustment (e.g., Schwartz et al., 2005; Juvonen et al., 2011; Takizawa et al., 2014). In addition to their influence on social and economic outcome variables, experiences of peer victimization seem to significantly increase the risk of experiencing various mental disorders like depression, anxiety disorders, PTSD, or substance abuse in childhood and adulthood (e.g., Stapinski et al., 2014; Hébert et al., 2016; Earnshaw et al., 2017). Moreover, a longitudinal study showed an association between frequent victimization and suicide attempts and suicide for girls later in life (Klomek et al., 2009). Thus, the experience of peer victimization in childhood and adolescence has long-term consequences that have a particular impact on mental health even decades after the exposure. Following on from this, studies indicate that experiences of adverse experiences in childhood and adolescence are related to an altered stress response, or structural and functional brain changes which in turn may have an impact on mental health in adulthood (Brendgen et al., 2017; Aults et al., 2019; Quinlan et al., 2020). In addition to physiological factors, altered attentional processes, or attentional biases, have been discussed in the context of traumatic childhood experiences and psychopathology in later life (Fani et al., 2011; Günther et al., 2015; Kelly et al., 2015; Iffland et al., 2019).

Attentional biases refer to the altered attentional focus on stimuli, are influenced by the valence or relevance of a stimulus, and are shaped by individual factors, such as emotional states or psychopathological symptoms (Koster et al., 2004, 2005; Bar-Haim et al., 2007; Cisler and Koster, 2010; Hankin et al., 2010; Peckham et al., 2010). Since they can influence perception and interpretation, and thus cognition and behavior, attentional biases are therefore considered in theories of the development and maintenance of mental disorders (for a review see Cisler and Koster, 2010). Investigating attentional biases in more detail, three different forms of attentional bias can be distinguished (i.e., facilitated attention, difficulties of disengagement, and attentional avoidance; Koster et al., 2004; Cisler and Koster, 2010). Facilitated attention is reflected in the way that emotional stimuli attract attention and thus attention is shifted to these stimuli more quickly. Difficulties in disengagement refer to the extent to which a stimulus attracts attention. This is accompanied by the difficulty in shifting attention from one stimulus to another stimulus. Attentional avoidance is manifested by the avoidance of shifting attention to potentially threatening stimuli and instead directing it to stimuli that are not threatening (Koster et al., 2004; Cisler and Koster, 2010).

Attentional biases have robustly been shown in individuals with depression or various forms of anxiety disorders (for a detailed overview see Bar-Haim et al., 2007; Cisler and Koster, 2010; Peckham et al., 2010). Furthermore, attentional biases have been repeatedly reported in victims of abuse and neglect with and without psychopathology (e.g., Pine et al., 2005; Fani et al., 2011; Romens and Pollak, 2012; Günther et al., 2015; Kelly et al., 2015). Previous research has suggested that experiences of maltreatment appear to influence attentional processes mainly in response to threatening stimuli (Gibb et al., 2009; Iffland and Neuner, 2020). In these studies, abused children had a higher tendency to attend to threatening stimuli, had problems shifting their attention away from cues of anger, and were faster in recognizing anger with less information (for a detailed overview see Jaffee, 2017). For example, using an emotional Stroop task and a dot-probe task, Iffland and Neuner (2022) found that emotional abuse was a significant predictor of attentional biases toward negatively associated neutral faces. In the dot-probe task, this was reflected in facilitated attention to these facial stimuli (Iffland and Neuner, 2022). Other studies found attention avoidance of threatening stimuli associated with child maltreatment (Pine et al., 2005; Kelly et al., 2015). With respect to studies of attentional biases in depression, there is evidence that altered attention allocation in the context of maltreatment does not only refer to a potential threat (Romens and Pollak, 2012; Günther et al., 2015). Günther et al. (2015) examined the connection between child maltreatment experiences and attentional processes using a dot-probe task in adults with a diagnosis of major depression. The authors found that experiences of child maltreatment were associated with altered attention allocation to sad facial expressions. This result was independent of symptom severity. Sustained attention toward sad faces was shown to be a stronger mood-congruent bias in depressed individuals with a history of child maltreatment (Günther et al., 2015). However, there have also been studies that found no evidence of attentional biases to emotional stimuli in general or to negative stimuli when analyzing reaction times within the dot-probe task in maltreated individuals (Fani et al., 2011; Hoepfel et al., 2022). Thus, although the results are not entirely conclusive, there is substantial evidence of attentional bias in the context of child maltreatment experiences.

Similar to child maltreatment perpetrated by adults or caregivers, relational peer victimization was associated with altered attention processes. Particularly, peer abused children showed less interference when confronted with victim-related words in an emotional Stroop task (Rosen et al., 2007). In another study examining adult psychiatric patients and healthy controls, Iffland et al. (2019) reported attentional biases in individuals who experienced relational peer victimization. Independent of the presence of mental illness, peer victimized individuals showed attentional avoidance in response to emotional words. Notably, avoidance was found not only in response to threatening stimuli but to emotional stimuli in general. In addition, Iffland and Neuner (2022) identified attentional biases for neutral faces previously conditioned with negative stimuli in individuals with experiences of relational peer victimization. Specifically, retrospective reports of relational peer victimization made an incremental contribution to the prediction of attentional biases beyond child maltreatment. Yet, they found no evidence of attentional avoidance, but rather an attentional bias toward threatening stimuli (Iffland and Neuner, 2022). Hence, regarding the impact of experiences of peer victimization on attention allocation, findings are not entirely conclusive as they have differed concerning the type of attentional biases and the valence of the stimuli for which biases occur.

To extend existing knowledge regarding the association between relational peer victimization and attentional processes we conducted an online facial dot-probe task (MacLeod et al., 1986) with emotional faces. The present study sought to investigate the influence of relational peer victimization experiences in childhood and adolescence on attentional processes and biases when using stimuli that are relevant in social interactions (emotional facial expressions). Attentional processes were examined in relation to positive and negative stimuli, with negative stimuli distinguished between negative, non-threatening stimuli (sad faces) and potentially threatening stimuli associated with victimization experiences (angry and disgusted faces). Social threat and exclusion are communicated not only through angry facial expressions but also through disgusted facial expressions, as there is an interpersonal aspect of disgust that is elicited by undesirable individuals to protect the social order (Rozin et al., 2008; Tybur et al., 2013). This more nuanced stimulus selection including potentially threatening and non-threatening negative emotions was used to provide a more accurate analysis of attentional processes that extends the findings from previous dot-probe studies in peer-victimized adults (Iffland et al., 2019; Iffland and Neuner, 2022). Drawing from the findings of previous research (Iffland and Neuner, 2022), relational peer victimization was expected to make a significant contribution to the prediction of attentional biases beyond the influence of child maltreatment experiences. We assumed that this would be particularly the case for emotions that are relevant in the context of peer victimization (i.e., anger, disgust). Based on previous results (Iffland and Neuner, 2022) we expected attentional biases to be evident in heightened attention to potentially threatening stimuli.

## 2. Materials and methods

### 2.1. Participants

Participants were recruited through the distribution of the participation link or QR code with access to the study *via* social media and flyers. In addition, patients receiving care at two outpatient clinics [*Bielefelder Institut für Psychologische Psychotherapieausbildung* (BIPP) and *Psychotherapeutische Ambulanz der Universität Bielefeld* (PAdUB)] were recruited for participation. The flyer contained information about the aims and methods of the study as well as a notice about the anonymity of the participation. At the beginning of the experiment information on general sociodemographic variables such as age, gender, educational level, and family status were requested. Furthermore, the instrument assessed the presence of mental illnesses, the use of medication, and other physical and psychological health questions in addition to the questionnaires used in this study. The sociodemographic and psychopathological characteristics of the 90 participants who were included in the analyses can be found in Table 1.

**Table 1:** Subject soziodemographic and psychopathological characteristics (*N* = 90).

**Characteristics**	
Gender, % female (*n*)	80.0 (72)
Age, *M* (*SD*)	28.8 (11.1)
Family status, % single (*n*)	38.9 (35)
Educational status (high school or higher), % (*n*)	91.1 (82)
Mental disorder in the past/currently^*a*^, % (*n*)	45.6 (41)/30.0 (27)
Symptoms of depression^*b*^, *M* (*SD*)	14.9 (11.0)
Psychopathology^*c*^, *M* (*SD*)	19.9 (17.2)
Trait anxiety^*d*^, *M* (*SD*)	44.9 (13.2)
Child maltreatment experiences^*e*^, *M* (*SD*)	40.1 (15.0)
Emotional abuse, *M* (*SD*)	10.1 (4.6)
Emotional neglect, *M* (*SD*)	10.1 (4.6)
Physical abuse, *M* (*SD*)	6.3 (2.5)
Physical neglect, *M* (*SD*)	7.1 (2.7)
Sexual abuse, *M* (*SD*)	6.6 (3.8)
Minimization/denial, *M* (*SD*)	0.4 (0.8)
Peer victimization experiences^*f*^, *M* (*SD*)	10.3 (7.5)

^*a*^based on self-report; ^*b*^Beck Depression Inventory; ^*c*^Symptom Checklist-27; ^*d*^State Trait Anxiety Inventory (Trait); ^*e*^Childhood trauma questionnaire; ^*f*^Fragebogen zu belastenden Sozialerfahrungen.

### 2.2. Procedure

Questionnaires were administered using the Qualtrics survey platform. For the experiment, the web version of the program Inquisit 6 (Millisecond software) was used. At the beginning of the study, participants were informed that participation was voluntary and that it was possible to quit the study at any time without penalty. They were also informed that participation would not be remunerated and that confidential information about mental health symptoms and stressful life experiences would be collected. Participation was only possible after participants had given their consent to participate by clicking on a box. After answering the questionnaires, participants were redirected to the Inquisit homepage, from where the Inquisit application could be downloaded. The experiment was designed in such a way that it could be carried out on both computers and mobile devices. The procedure was approved by the Ethics Committee of Bielefeld University.

### 2.3. Symptoms of psychopathology

To assess general symptoms of psychopathology the Symptom Check List-27 (SCL-27; Hardt and Gerbershagen, 2001) was used. This 27-item questionnaire captures different areas of psychological symptoms (six subscales including depressive, dysthymic, vegetative, agoraphobic, sociophobe symptoms, and symptoms of mistrust). For this sample, there was an excellent internal consistency (Cronbach's α = 0.94).

The German version of the Beck Depression Inventory (BDI II; Hautzinger et al., 2006; Kühner et al., 2007) was used to assess current depressive symptomatology over the last 2 weeks. This questionnaire uses 21 items to assess the severity of depressive symptoms on a scale from zero (absent) to four (severely present). The sum value of the items allows conclusions to be drawn about the severity of depressive symptoms (no/minimal, mild, moderate, or severe depressive symptoms). In the present sample, the BDI II showed excellent internal consistency (Cronbach's α = 0.94).

The trait subscale of the State-Trait-Anxiety-questionnaire (STAI; Spielberger et al., 1970; Laux et al., 1981) was used to measure trait anxiety. This subscale measures anxiety as a trait by using 20 items rated on a scale from one (almost never) to four (almost always). The STAI showed excellent internal consistency for the present sample (Cronbach's α = 0.95).

### 2.4. Experiences of maltreatment and peer victimization

Experiences of relational peer victimization were assessed by using the *Fragebogen zu belastenden Sozialerfahrungen* (FBS, Adverse Social Experiences Quesstionnaire; Sansen et al., 2013). This questionnaire retrospectively assesses experiences of various forms of relational peer victimization, distinguishing experiences that occurred during childhood (age 6–12) and adolescence (age 13–18) by using 22 items asking about whether a specific social situation was experienced or not. In the present sample, the FBS showed excellent internal consistency (Cronbach's α = 0.90). Although the FBS consists of two subscales (separating experiences in childhood and adolescence) it is recommended to use the total score, as there is evidence that it is superior to the subscales in capturing stressful social experiences (Sansen et al., 2013).

For examining experiences of child maltreatment, the German version of the Childhood Trauma Questionnaire (CTQ; Wingenfeld et al., 2010) was used to retrospectively assess different forms of child maltreatment experiences. The CTQ consists of 28 items on five subscales (physical maltreatment, physical neglect, emotional maltreatment, emotional neglect, and sexual abuse). For the total number of items, the CTQ in our sample showed excellent internal consistency (Cronbach's α = 0.90). For the CTQ subscales of emotional abuse, emotional neglect, physical abuse, and sexual abuse internal consistency was acceptable to excellent (all α >0.79). As found in previous research (Klinitzke et al., 2012) the physical neglect subscale demonstrated only a questionable internal consistency (Cronbach's α = 0.60). In addition to the five subscales, the CTQ captures the tendency to underreport maltreatment experiences with the minimization/denial scale (three items). Values above zero indicate response bias (false negatives) (Bernstein et al., 1994).

### 2.5. Paradigm and stimuli

For measuring attentional biases, the facial dot-probe paradigm (MacLeod et al., 1986) was used. A fixation cross was presented in the center of the screen for 500 ms. This was followed by the simultaneous and horizontal presentation of two still images for 500 ms. In 80% of the trials, one of the images was an emotional face and the other was a neutral face. In 20% of the trials, both images were neutral. Then a gray dot appeared on one of the two sides of the screen and replaced one of the two images. In congruent trials, the dot replaced the emotional face, in incongruent trials the dot replaced the neutral one. The participants were asked to indicate as quickly and accurately as possible if the dot was presented either on the right or the left side of the screen (by pressing the key ‘E' for left and the key ‘I' for right on the desktop version of the experiment or by clicking on the right/left side of the screen in the mobile version of the experiment). Four different emotional facial expressions (sad, happy, angry, and disgusted) and neutral facial expressions were used. In addition to the emotional-neutral trials, there were also neutral-neutral trials serving as baseline trials for measuring the different kinds of attentional bias scores. A total of 50 different pictures of 10 actors (five men, five women) were taken from the Radboud Faces Database (Langner et al., 2010). Each actor with each emotion was presented twice. Accordingly, the neutral images were presented more often. The order of trials and the selection of the individual emotions were randomized.

### 2.6. Data reduction

Drawing from previous studies, the reaction time data were adjusted in several steps (Koster et al., 2004; Bardel et al., 2013; Iffland and Neuner, 2022). Trials in which the location of the dot was incorrectly reported were removed from the trials to be analyzed (1.3% of all trials). No participant had an error rate higher than 25%. In addition, all trials in which subjects had a reaction time of <150 ms or more than 2,000 ms were not included in the analyses (0.1% of all trials). Moreover, individuals whose mean reaction time deviated more than 3 SD from the sample mean reaction time were excluded from the analyses (*n* = 1). In addition, individual trials were removed in which the reaction time deviated more or less than 2 SD from the individual mean reaction time (4.4% of all trials). For measuring the attentional bias scores for each trial type (angry-neutral, sad-neutral, disgust-neutral, happy-neutral) the overall attentional bias score was calculated by subtracting the reaction times for congruent trials (i.e., trials in which the dot replaced the emotional face) from the reaction time for incongruent trials (i.e., trials in which the dot replaced the neutral face). Attention biases are reflected in shorter reaction times for the dot when attention was focused on this area and longer reaction times for the dot when attention was not focused there. Based on the calculation of the score, positive values for the attentional bias score indicated that the attention was on the emotional faces, whereas negative values indicated that the attention of the subjects was not on the emotional face, but the neutral face. To specify altered attentional processes more precisely with respect to the different types of attentional biases, the orientation score and the disengaging score were calculated in addition to the attentional bias score to capture processes of facilitated attention or difficulties of disengagement (Koster et al., 2004). For calculating the orienting score, the reaction time for congruent trials was subtracted from the reaction time for trials in which two neutral faces were presented. This score provides information about whether subjects shifted their attention more quickly to the emotional stimulus. In addition, to gain information about whether subjects had difficulty shifting their attention away from the emotional stimuli, the disengaging score was calculated. For this purpose, reaction times in trials in which two neutral pictures were presented were subtracted from reaction times in incongruent trials.

### 2.7. Statistical analyses

For sample size estimation a statistical power analysis was calculated for the multiple regression analyses using G Power 3.1 (Faul et al., 2009). Based on previous results (Günther et al., 2015; Iffland and Neuner, 2020, 2022) a medium to large effect size (Cohen, 1988) was assumed (Cohen's *f*^2^ = 0.25). Thus, with α = 0.05, power = 0.95, and the initially planned inclusion of seven predictors (age, psychopathology, emotional abuse, emotional neglect, physical abuse, sexual abuse, peer victimization) the required sample size was *N* = 86.

Statistical analyses were performed using the Statistical Package for the Social Sciences (IBM SPSS Statistics 28). For all analyses, a significance level of *p* ≤ 0.05 was used. Correlation analyses and *t*-tests were adjusted for multiple comparisons using false discovery rate (FDR) correction (Benjamini and Hochberg, 1995). To calculate the influence of peer victimization on the different attentional bias scores and to control for the influence of child maltreatment experiences, several sets of hierarchical multiple regression analyses were calculated. Two subscales of the CTQ were not included in the analyses: the sexual abuse subscale due to a lack of variance in our sample, and the physical neglect subscale due to the weak internal consistency and high intercorrelations with other subscales (Klinitzke et al., 2012). For completeness and comparability, the two subscales were nevertheless included in the descriptive statistics. FDR-adjusted Pearson correlation coefficients of peer victimization, the three subscales of child maltreatment, and psychopathological measures are shown in Table 2. To control for the influence of symptoms of psychopathology and age of the participants, the first step of all regression models included the sum score of the SCL-27 and age. Due to the high correlation of the SCL-27 scores with the BDI II scores (*r* = 0.84, *p* < 0.001) and the STAI scores (*r* = 0.76, *p* < 0.001), only the SCL-27 was included in the regression analyses. In a second step, the sum scores of the CTQ subscales were included in the model (i.e., subscales of emotional abuse, emotional neglect, and physical abuse). In a final step, the FBS sum score (i.e., peer victimization) was included as the last predictor in the model. These regression analyses were conducted separately for the individual bias indices and the respective emotion presented. Participants who scored on all three items of the minimization/denial subscale of the CTQ (*n* = 3) were excluded from the analyses (Iffland et al., 2013; Ross et al., 2019). As the pattern of results did not change, the results reported refer to the whole sample. Analyses showed no violation of the multicollinearity assumption (all tolerances ≥ 0.31; all variance inflation factors ≤ 3.28).

**Table 2:** Pearson correlation coefficients of peer victimization and the different types of child maltreatment experiences and psychopathological measures.

**Trial type**	**Peer victimization^*a*^**	**Emotional abuse^*b*^**	**Emotional neglect^*b*^**	**Physical abuse^*b*^**
	***r***	***r***	***r***	***r***
Peer victimization	-	-	-	-
Emotional abuse	0.40***	-	-	-
Emotional neglect	0.41***	0.79***	-	-
Physical abuse	0.28**	0.62***	0.52**	-
Psychopathology ^*c*^	0.54***	0.46***	0.47***	0.33**
Trait anxiety ^*d*^	0.42***	0.56***	0.52**	0.27*
Symptoms of depression^*e*^	0.44***	0.46***	0.45***	0.21*

*p < 0.05, **p < 0.01, and ***p < 0.001; *p* values are FDR-adjusted; ^*a*^Fragebogen zu belastenden Sozialerfahrungen; ^*b*^Childhood trauma questionnaire; ^*c*^Symptom Checklist-27; ^*d*^State trait anxiety inventory (Trait); ^*e*^Beck depression inventory.

## 3. Results

A detailed description of the attentional bias index scores for the different emotions, the mean values, standard deviations, and one-sample *t*-tests of the absolute index scores for the presentation of one emotion each are shown in Table 3. The results of the *t*-tests showed that the orienting scores for disgusted and happy faces differed significantly from zero and were negative, indicating attentional avoidance of happy and disgusted facial expressions.

**Table 3:** Results of one sample t-tests for the different index scores.

**Trial type**	***M* (*SD*)**	***t*(89)**	***p***	**Cohen's *d***
**Attentional bias score**				
Anger	1.67 (25.04)	0.63	0.634	|0.07|
Disgust	−5.63 (27.15)	−1.97	0.156	|0.21|
Sadness	1.87 (28.99)	0.61	0.591	|0.06|
Happiness	−4.81 (29.74)	−1.53	0.340	|0.16|
**Orienting score**				
Anger	−0.35 (21.45)	−0.16	0.877	|0.02|
Disgust	−7.20 (22.50)	−3.04	0.018*	|0.32|
Sadness	−3.53 (23.13)	−1.45	0.259	|0.15|
Happiness	−8.55 (25.65)	−3.16	0.024*	|0.33|
**Disengaging score**				
Anger	2.02 (22.42)	0.86	0.591	|0.09|
Disgust	1.57 (20.34)	0.73	0.621	|0.08|
Sadness	5.40 (25.89)	1.98	0.204	|0.21|
Happiness	3.74 (24.21)	1.47	0.292	|0.16|

*p < 0.05; *p* values are FDR-adjusted.

The bivariate Pearson correlation coefficients between maltreatment experiences and the different index scores for each trial type can be found in Table 4. The correlations between the FBS sum score and the different index scores for each emotion were not significant (all FDR corrected *p*'s > 0.05). The analyses showed a positive correlation between the emotional abuse score and the disengaging score for sad-neutral trials as well as a positive correlation between the emotional neglect score and the disengaging score for sad-neutral trials.

**Table 4:** Pearson correlation coefficients of the different types of maltreatment experiences and the index scores for each trialtype.

**Trial type**	**Peer victimization^*a*^**	**Emotional abuse^*b*^**	**Emotional neglect^*b*^**	**Physical abuse^*b*^**
	***r***	***r***	***r***	***r***
**Attentional bias score**				
Anger	−0.07	0.03	−0.09	−0.04
Disgust	0.01	−0.16	−0.21	−0.09
Sadness	0.18	0.19	0.13	0.15
Happiness	−0.21	−0.13	−0.05	−0.02
**Orienting score**				
Anger	0.02	0.14	0.02	0.00
Disgust	0.01	−0.09	−0.12	−0.05
Sadness	0.21	−0.13	−0.18	−0.04
Happiness	−0.10	−0.14	−0.02	−0.03
**Disengaging score**				
Anger	−0.10	−0.10	−0.12	−0.04
Disgust	−0.01	−0.11	−0.15	−0.07
Sadness	0.02	0.32*	0.31*	0.19
Happiness	−0.15	−0.01	−0.04	0.00

*p < 0.05; *p* values are FDR-adjusted; ^*a*^Fragebogen zu belastenden Sozialerfahrungen; ^*b*^Childhood trauma questionnaire.

### 3.1. Peer victimization

The hierarchical regression analyses for sad-neutral trials are presented in Table 5. Analyses showed that peer victimization made a significant contribution of variance in the prediction of the orienting score for sad faces. Here, peer victimization was not only the strongest predictor but also the only one with a significant positive association with the orienting score for sad faces. Higher scores on the FBS, and thus more reported peer victimization experiences, were associated with higher scores on the orienting score in the present sample. By including this predictor in the third step, the contribution to variance was 8% [final model: *F*_(6, 83)_ = 2.34, adjusted *R*^2^ = 0.08, *p* = 0.039]. There was no significant relationship between the index scores and the level of peer victimization experiences for angry faces (see Table 6). Similarly, no significant effects were found in response to disgusted faces for the attentional bias score [final model *F*_(6, 83)_ = 0.91, adjusted *R*^2^ = –0.01, *p* = 0.494], the orienting score [final model: *F*_(6, 83)_ = 0.62, adjusted *R*^2^ = –0.03, *p* = 0.713] and the disengaging score [final model: *F*_(6, 83)_ = 1.47, adjusted *R*^2^ = 0.03, *p* = 0.197]. In addition, there were no significant effects for trials in which happy facial expressions were presented for the attentional bias score, the orienting score, and the disengaging score (see Table 7).

**Table 5:** Hierarchical multiple regression analyses for sad-neutral trials.

**Variable**	**β**	***R* ^2^**	**Adjusted *R*^2^**	**△*R*^2^**	***F***
**Attentional bias score**					
Step 1		0.03	0.01	0.03	1.34
Age	−0.06				
SCL-27	0.04				
Step 2		0.05	−0.01	0.02	0.88
Emotional abuse	0.15				
Emotional neglect	−0.07				
Physical abuse	0.04				
Step 3		0.06	−0.01	0.01	0.84
Peer victimization	0.11				
**Orienting score**					
Step 1		0.03	0.01	0.03	1.35
Age	−0.10				
SCL-27	−0.02				
Step 2		0.07	0.02	0.04	1.29
Emotional abuse	−0.08				
Emotional neglect	−0.27				
Physical abuse	0.07				
Step 3		0.15	0.08	0.08	2.34*
Peer victimization	0.33**				
**Disengaging score**					
Step 1		0.03	0.01	0.03	1.20
Age	0.02				
SCL-27	0.06				
Step 2		0.12	0.06	0.09	2.18
Emotional abuse	0.25				
Emotional neglect	0.17				
Physical abuse	−0.02				
Step 3		0.14	0.07	0.02	2.18
Peer victimization	−0.18				

*p < 0.05, **p < 0.01; β coeffizients correspond to those of the final model.

**Table 6:** Hierarchical multiple regression analyses for anger-neutral trials.

**Variable**	**β**	***R* ^2^**	**Adjusted *R*^2^**	**△*R*^2^**	***F***
**Attentional bias score**					
Step 1		0.12	0.10	0.12	5.67**
Age	0.37***				
SCL-27	−0.13				
Step 2		0.18	0.13	0.06	3.68**
Emotional abuse	0.42*				
Emotional neglect	−0.40*				
Physical abuse	−0.08				
Step 3		0.18	0.12	0.00	3.08**
Peer victimization	0.06				
**Orienting score**					
Step 1		0.04	0.01	0.04	1.58
Age	0.10				
SCL-27	−0.32*				
Step 2		0.11	0.06	0.08	2.12
Emotional abuse	0.45*				
Emotional neglect	−0.19				
Physical abuse	−0.12				
Step 3		0.13	0.06	0.01	1.97
Peer victimization	0.14				
**Disengaging score**					
Step 1		0.08	0.06	0.08	3.58*
Age	0.31**				
SCL-27	0.16				
Step 2		0.12	0.06	0.04	2.20
Emotional abuse	0.04				
Emotional neglect	−0.26				
Physical abuse	0.03				
Step 3		0.12	0.05	0.00	1.85
Peer victimization	−0.06				

*p < 0.05, **p < 0.01, and ***p < 0.001; β coeffizients correspond to those of the final model.

**Table 7:** Hierarchical multiple regression analyses for happy-neutral trials.

**Variable**	**β**	***R* ^2^**	**Adjusted *R*^2^**	**△*R*^2^**	***F***
**Attentional bias score**					
Step 1		0.05	0.03	0.05	2.16
Age	−0.23*				
SCL-27	−0.06				
Step 2		0.08	0.02	0.03	1.42
Emotional abuse	−0.29				
Emotional neglect	0.28				
Physical abuse	0.10				
Step 3		0.11	0.05	0.03	1.77
Peer victimization	−0.23				
**Orienting score**					
Step 1		0.06	0.04	0.06	2.81
Age	−.21				
SCL-27	−.23				
Step 2		0.12	0.07	0.06	2.24
Emotional abuse	−0.38*				
Emotional neglect	0.38*				
Physical abuse	0.10				
Step 3		0.12	0.06	0.00	1.86
Peer victimization	−0.03				
**Disengaging score**					
Step 1		0.01	−0.02	0.01	0.21
Age	−0.06				
SCL-27	0.17				
Step 2		0.01	−0.05	0.00	0.16
Emotional abuse	0.05				
Emotional neglect	−0.06				
Physical abuse	0.02				
Step 3		0.05	−0.02	0.04	0.74
Peer victimization	−0.25				

*p < 0.05; β coeffizients correspond to those of the final model.

### 3.2. Further analyses of child maltreatment

Regarding the prediction of attentional biases in angry-neutral trials (see Table 6), the regression models showed that experiences of emotional abuse and emotional neglect were, besides age, the only significant predictors in the final regression model [*F*_(6, 83)_ = 3.08, adjusted *R*^2^ = 0.12, *p* = 0.009]. The associations with the attentional bias score behaved in opposite ways. Higher scores on the emotional abuse subscale were associated with higher attentional bias scores, whereas higher scores on emotional neglect were associated with lower attentional bias scores on angry faces. For the orienting score, emotional abuse was a significant predictor, with an overall non-significant final model [final model: *F*_(6, 83)_ = 1.97, adjusted *R*^2^ = 0.06, *p* = 0.079]. Associations between emotional maltreatment experiences and the orienting score for happy faces could also be found in happy-neutral trials (see Table 7). This relationship was inverse to that found for angry faces. Emotional abuse experiences were associated here with lower scores and emotional neglect with higher scores for happy faces. However, the overall model for the orienting score in happy-neutral trials was not significant [final model: *F*_(6, 83)_ = 1.86, adjusted *R*^2^ = 0.06, *p* = 0.098].

## 4. Discussion

Given the ambiguous findings on attentional biases in the context of peer victimization, the present work provided new insights into the relationship between relational peer victimization and attentional biases beyond the influence of child maltreatment experiences. In this context, the present study was designed to provide differentiated accounts of attentional biases in the context of maltreatment and peer victimization experiences, thus extending previous research. Consistent with our hypothesis we found altered attentional processes in individuals reporting higher levels of victimization experiences in the present sample. However, this influence was found in sad faces and not, as previously hypothesized, in emotions that were expected to be relevant as threatening stimuli in the context of peer victimization. Furthermore, altered attention processes were found in individuals reporting experiences of emotional maltreatment when confronted with angry facial expressions.

In the present study, the results indicated evidence for facilitated attention to sad facial expressions in individuals with higher levels of relational peer victimization experiences beyond the influence of experiences of child maltreatment. This effect was seen even when controlling for symptoms of psychopathology. These findings were consistent with the results of Günther et al. (2015), who also found facilitated attentional orienting to sad faces in depressed individuals with experiences of child maltreatment when controlling for depressive symptoms. Previous research suggested that attentional biases not only manifest in biased attention regarding potentially threatening stimuli but could also be influenced by a person's mood or are mood-congruent (Koster et al., 2005; Hankin et al., 2010; Romens and Pollak, 2012; Günther et al., 2015). Similarly, previous research revealed the existence of attentional biases in currently depressed or at-risk children and adolescents (e.g., Joormann et al., 2007; Hankin et al., 2010). Accordingly, the presentation of faces in the current study may have triggered negative emotions associated with social interactions, which may have facilitated processing of sad stimuli. In line with this argument, previous research has emphasized the relevance of sadness in the context of victimization and maltreatment. Victims of bullying tend to be insecure and fearful and they are more likely to have a negative view of themselves and rate themselves as stupid or flawed (Olweus, 1994). In a study by Mahady Wilton et al. (2000) the authors observed the behavior of elementary school children and found signs of sadness significantly more often in victims of bullying than in perpetrators, which could be related to the perceived failures in achieving one's goals in social situations. The authors note that sadness signals to the perpetrator that his goal of causing suffering is being met and thus becomes reinforcing, increasing the likelihood of becoming a victim (Mahady Wilton et al., 2000). In addition, previous studies found increased self-reported sadness among victims of bullying (Camodeca and Goossens, 2005; Glew et al., 2005). However, heightened attention for sadness cues is not exclusively associated with experiences of peer victimization. Romens and Pollak (2012) used a mood induction before a dot-probe task with depression-relevant cues and found that children with experiences of physical abuse showed heightened attention for these cues after the induction of sadness. This result is in line with various other studies showing altered responses on a behavioral and neural level in studies using sad faces in participants with various forms of traumatic childhood experiences (for a review see Saarinen et al., 2021). Hence, findings of an altered reaction to sad facial expressions in the wake of peer victimization in the present study may also apply to adverse childhood experiences in general. In conjunction with evidence of mood-congruent bias in depression and at-risk depression (e.g., Joormann et al., 2007; Hankin et al., 2010), the present findings may be indicative of biased information processing in peer victimized individuals that may be relevant in putting individuals at risk for the development of psychopathology. Following Rosen et al. (2007), victims may implicitly associate themselves with victimization which decisively influences cognitions, behavior, and emotions in future social situations.

There was no influence of peer victimization on participant scores when angry or disgusted faces were presented. Further, there was no significant influence of peer victimization on reaction times for happy faces although the results of the one sample *t*-tests suggest that participants generally showed significant avoidance of happy and disgusted faces. Therefore, the findings for the overall sample are not reflected in the analyses for peer victimization. The present results contradict the findings of Iffland et al. (2019) and Iffland and Neuner (2022) who found a significant contribution of peer victimization experiences for attentional biases for potentially threatening stimuli and positive emotional stimuli in their studies. Using a social conditioning task or social evaluative words in these studies, the participants presumably established a reference to themselves in terms of the potentially threatening nature of the stimuli, which may have significantly influenced attentional processes. The simple presentation of emotional faces used in the present study may not activate the social victim schema in a way that leads to higher vigilance for threatening stimuli or emotional stimuli in general. This may lead to the finding that emotions, which were expected to be relevant in the context of peer victimization, were not associated with attentional biases here. We suspect that the mere presentation of emotional faces is more of a projection screen for one's emotional state. Since the results of Iffland et al. (2019) and Iffland and Neuner (2022) also point in different directions concerning the type of attentional biases, it can be assumed that stimulus choice is likely to be crucial for the presence and nature of attentional biases.

Furthermore, analyses showed altered attentional processes that were related to higher levels of emotional childhood maltreatment. These processes were particularly evident for angry faces and suggest that experiences of emotional abuse led to increased attention toward angry faces. The results confirmed the assumption that attention processing of angry faces as potentially threatening stimuli is influenced by adverse childhood experiences (Gibb et al., 2009; Kelly et al., 2015; Iffland and Neuner, 2020). In addition, our findings indicated attentional avoidance of happy facial expressions in individuals reporting emotional abuse experiences, which may be indicative of dysfunctional emotion regulation. In support of this hypothesis, Burns et al. (2010) showed that experiences of emotional abuse were associated with difficulties in emotion regulation. In contrast to experiences of emotional abuse, our results showed that emotional neglect was associated with avoidance of angry faces. These differentiated findings for different subtypes of maltreatment experiences are in line with the results of Iffland and Neuner (2020) who used a face in the crowd task in their study to highlight that attentional processes differ between the two forms of emotional maltreatment. They reported a faster detection of negative faces in victims of emotional abuse, whereas slower recognition of negative and neutral faces was more likely in victims of emotional neglect. However, in contrast to the findings of Iffland and Neuner (2020), our findings regarding emotional neglect are less indicative of a general avoidance of emotional faces than of more differentiated processes, possibly involving the avoidance of highly salient stimuli (here angry faces). This is supported by the finding that emotional neglect was associated with an attentional shift toward happy faces. These findings could thus be the result of emotion regulation strategies, which in the context of emotional neglect could be associated with avoidance of threatening stimuli and a shift toward positive stimuli. However, because the final regression model for the orienting score for happy-neutral trials did not reach significance, these conclusions must be viewed with caution. Against our expectations, there was no relationship between maltreatment experiences and reaction times for disgust-neutral trials. Horstmann (2003) showed that disgusted facial expressions are more likely to be interpreted as expressions of emotional experience, whereas anger is more likely to be perceived as having an informative character at the interpersonal level. Future studies should therefore consider the use of disapproval faces as stimuli for social rejection (Burklund et al., 2007). Our results indicate differentiated attentional processes that are influenced by various forms of maltreatment, but particularly in individuals with emotional or relational maltreatment experiences.

### 4.1. Limitations

Limitations of the present study must be considered when interpreting the results. One-third of the participants stated that they were currently suffering from a mental disorder. For reasons of anonymity, no information could be collected on whether participants were patients of the outpatient clinics. It cannot be excluded that treatment or current medication influenced the attentional processes or reaction times. However, by including psychopathological symptom severity in the regression models, and by adjusting the reaction time data, we were able to reduce the potential influence on our results. In addition, the data did not allow us to assert causal relationships due to the cross-sectional design of our study. Longitudinal studies for analyzing the relationship to mental health should be addressed in the future. The retrospective assessment of experiences of child maltreatment and peer victimization as self-reports also limits the interpretability of the results as they may be affected by distortions (Baldwin et al., 2019). However, the questionnaires used in the present study have repeatedly shown good reliability and validity and are therefore suitable for the retrospective recording of stressful life experiences (Klinitzke et al., 2012; Sansen et al., 2013). Another limitation of the study is the interpretation of reaction times when using the dot-probe task. However, the reliability can be increased by the experimental design of the dot-probe task, e.g., by choosing a horizontal instead of a vertical stimulus presentation (Price et al., 2015). Moreover, the findings indicated that the dot-probe paradigm was sensitive enough to allow differentiation between emotions. Nevertheless, our results should be interpreted with caution, especially since the one-sample *t*-tests for the absolute orienting scores are only significant for happy and disgusted facial expressions and not for sad and angry faces. In addition, the results should be interpreted with caution due to the low controllability of the entire study, caused by its realization as an online study. It should be noted that it was not possible to determine which device was used for participation. It cannot be ruled out that the type of device (computer or mobile device) had an influence on the results. In addition, the online study could not control the situational conditions under which the performance took place. However, by adjusting the experimental data, we were able to minimize the influence that the behavior would have had if the instructions had not been followed or if the subjects had been unfocused or distracted. Moreover, results were consistent with the findings of several studies that have used reaction times, and also neural measures (Günther et al., 2015; Saarinen et al., 2021). Future research should nevertheless consider additional measures such as physiological measures to be able to interpret the results of a dot-probe task more reliably. In this context, physiological measurements, and the analysis of event-related potentials could provide more accurate information about attentional processes, since cortical responses can be recorded and analyzed in the range of milliseconds. The analysis of reaction times is limited to a specific point in time (here 500 ms after stimulus onset). So, our results do not provide information about the course of the attentional process. It cannot be excluded that attention has already been shifted. Future studies should therefore include variable presentation durations in addition to physiological outcomes to capture different stages of the attentional process (Chapman et al., 2019). Furthermore, it should be noted that neutral facial expressions were chosen as baseline. This may have influenced the results, as there is some evidence that individuals with experience of maltreatment perceive neutral faces as negative (Pollak et al., 2000). Nevertheless, our results indicate differences in attentional processes with respect to negative and neutral facial expressions, yet future work could consider the use of calm faces instead of neutral faces (Kelly et al., 2015).

## 5. Conclusion

In line with previous results, our study showed that experiences of relational peer victimization and emotional child maltreatment in childhood and adolescence influence attentional processes in adulthood. Higher levels of peer victimization were associated with facilitated attention to sad facial expressions in our sample. The results are thus indicative of mood-congruent attentional biases in individuals who have experienced relational peer violence. In addition, altered attentional processes for angry faces were present in participants with higher levels of emotional child maltreatment experiences. Adverse childhood experiences, particularly experiences of emotional maltreatment and relational peer victimization, can thus be considered relevant to the development of cognitive schemata that continue to be activated in adulthood, and therefore can potentially influence new experiences, feelings, thoughts in social situations, and thus presumably mental health.

## Data availability statement

The raw data supporting the conclusions of this article will be made available by the authors, without undue reservation.

## Ethics statement

The studies involving human participants were reviewed and approved by Ethics Committee of Bielefeld University. Written informed consent was not provided because of the design as an online study. The participants gave their informed consent by clicking on a box.

## Author contributions

KB contributed to the conception and design of the work, the acquisition, analysis, interpretation of the data, drafted, revised, approved the manuscript, and ensures the accuracy and integrity of any part of the work. BI was the chief investigator for this study, contributed to the conception of the study, supervised data analyses, participated in the interpretation of the data, and critically revised the manuscript for important intellectual content. All authors contributed to the article and approved the submitted version.
